# Core outcomes for speech-language services in Ontario schools: a group concept mapping study and guiding framework

**DOI:** 10.1186/s12913-024-10821-7

**Published:** 2024-03-16

**Authors:** Peter T. Cahill, Mark A. Ferro, Stella Ng, Lyn S. Turkstra, Wenonah N. Campbell

**Affiliations:** 1 School of Rehabilitation Science, Institute of Applied Health Sciences, McMaster University, Room 403, 1400 Main Street West, Hamilton, ON L8S 1C7 Canada; 2 CanChild Centre for Childhood Disability Research, Hamilton, Canada; 3 School of Public Health Sciences, University of Waterloo, Waterloo, Canada; 4 Department of Speech-Language Pathology, University of Toronto, Toronto, Canada; 5 Centre for Interprofessional Education, University of Toronto, Toronto, Canada

**Keywords:** Speech-language therapy, Speech-language pathology, Schools, Core outcome set, Group concept mapping, Cluster analysis

## Abstract

**Background:**

Establishing the most important outcomes for school-based speech-language therapy is essential to guide future research and program evaluation for these services. Many health disciplines have developed core outcomes sets (COS) for this purpose. A COS encompasses the most important outcomes for particular health services as identified by appropriate interested parties. These interested parties usually represent health care providers and those with the health condition. In this paper, we report the development of a guiding framework for a COS for speech-language therapy services in schools in a Canadian context.

**Methods:**

Using a group concept mapping method, we identified the outcomes for inclusion in the COS guiding framework through the elicited opinions of key interested parties: speech-language therapists, teachers, and family members of children with speech, language, and communication needs. We extracted 103 statements (potential outcomes) from a previous data set of interview transcripts. We then asked participants to sort the statements into conceptually similar groups, which were aggregated and transformed into a cluster map using multidimensional scaling followed by hierarchical cluster analysis. Participants also rated each statement on 5-point scales for importance and feasibility. We calculated mean ratings for individual statements and for all statements in a cluster, for all participants and for participant groups separately.

**Results:**

We identified seven core outcomes for school-based speech-language services in Ontario, Canada. These included: classroom-based services, a holistic approach, support for teachers, care coordination, accessible services, family supports, and student success. All outcomes were rated highly for importance. Feasibility ratings were consistently below importance ratings. All participant groups concurred that a holistic approach was the most important outcome and accessible services was the least feasible outcome to achieve.

**Conclusions:**

The seven outcomes identified in this study are recommended to guide the development of a full COS to direct future research and program evaluation for school-based speech-language services. These outcomes have not been widely included in previous research and should be incorporated into future research alongside specific intervention outcomes. Data for some outcomes may be available from non-traditional sources such as administrative data sets. Consequently, their use for program evaluations should be accompanied by appropriate institutional support to allow speech-language therapists to make meaningful use of appropriate outcomes data.

**Supplementary Information:**

The online version contains supplementary material available at 10.1186/s12913-024-10821-7.

## Background

The systematic collection and careful, proactive interpretation of outcomes – the results of care – provides the basis for evaluating and improving quality care [1–3]. Outcomes are evaluated using specific measures or target data points that are referred to as *indicators* [4–6]. Indicators are necessarily limited in scope, able to capture only a portion of the broader outcome [6]. For example, standardized scores from a language assessment battery would be an indicator for the outcome of expressive and receptive language abilities but would not capture all of the child’s language skills. Although a standardized language assessment cannot possibly tap into all language skills, scores from such an assessment may prove useful in making inferences about the broader skill of expressive or receptive language. Although indicators are supposed to provide meaningful information relevant to health care service quality and evaluation, indicators vary greatly in quality, relevance, and feasibility, and their selection requires complex, contextualized, systems-informed thinking [4–6]. Consequently, the careful selection of outcomes for healthcare services, as well as their associated indicators, is essential for supporting high quality care.

Within the field of speech-language therapy (SLT), robust and proactive outcomes use remains elusive [7–11]. In practice, successful outcomes collection depends on clinicians perceiving a need to evaluate an outcome that they value [12]. Further, it is important that patients and other interested parties have a voice in selecting outcomes for care [13] and for researchers to ensure that evidence is relevant [14–16]. Specifically, evidence regarding intervention effectiveness contained within the research literature would be more pertinent and provide more relevant information if it incorporated outcomes valued by patients and communities [16]. Similarly, the selection of indicators should be in consultation with those served by the health service [17].

One solution to ensure that outcomes and indicators are meaningful to interested parties is the creation of a Core Outcome Set (COS) wherein the most important and meaningful outcomes are selected through collaboration. For example, Morris et al. [18] collaboratively developed a COS for children with neurodevelopmental disabilities, in which interested parties (children, parents, and healthcare providers) recommended collecting patient-reported outcomes for communication, emotional wellbeing, pain, mobility, self-care, independence, community life, mental health, sleep, behaviour, safety, and toileting. Once a COS is developed, Crudgington and colleagues [19] recommended that it be used to document outcomes in clinical research and for tracking outcomes within clinical programs. However, less than 0.5% of developed COS have been within the field of rehabilitation [16], and most of those within physiotherapy. Therefore, it is relevant and timely to develop a COS to guide future work in the SLT profession.

Speech-language therapists (S-LTs) provide important supports to students in schools [20], including for children with diverse speech, language, and communication needs [21]. Practice in schools has long been recognized as particularly complex [22]. In recent years, models of school-based health services have evolved away from individual assessment and intensive pull-out intervention towards tiered models where the whole school community is offered several different service types that are matched to students’ needs [23, 24]. This evolution is thought to be motivated by a confluence of factors, including ever increasing demands for services in schools, growing recognition of historically underserved populations, intense resource allocation constraints, and a renewed focus on meaningful inclusion of children of all ability levels in social and academic life [25]. The shift to tiered models has occurred in Australia [26], Europe [23, 27], Canada [24, 28], and across the United States[29]. Several tiered models exist across jurisdictions, including Response to Intervention [23, 24, 30], Multi-Tiered Systems of Supports [29], and Partnering for Change [27, 31]. As part of their mandate to provide evidence-based services, S-LTs must make evidence-informed decisions regarding their choice of service delivery model [21].

Evidence to support decision-making about service delivery models is limited [30, 32, 33]. Multiple reviews have concluded that school-based S-LTs are not well supported by the current research evidence [32, 33]. Archibald [32] further recommended that clinicians evaluate the outcomes of their service delivery models, leaning on the traditions of program evaluation and quality improvement. Importantly, outcomes tracked in most SLT research do not generally match the types of outcomes that students and families identify as meaningful [34]. Nor are the outcomes consistent with those included in educational research into supporting these children, where educational access and success take prominence [35]. Previous theoretical work has suggested that outcomes beyond assessments of individual student abilities are relevant to making informed and meaningful decisions for services in schools [36, 37], an observation that empirical work has found to also be true of school-based practice [38]. Although previous reviews [32, 33] considered outcomes beyond individual student abilities, such as the capacities of teachers to support children with communication disorders in the classroom, the evidence they located was limited to a narrow list of individual student outcomes (e.g., phonological awareness, vocabulary). Consequently, it is timely to establish what outcomes are meaningful to interested parties to inform future research and clinical practice regarding school-based SLT. This would help address the mismatch between outcomes included in research studies and those that families and educational research appear to identify as important. Further, selecting outcomes is an important first step in evaluating services and programs [39]. Thus, developing a COS for tiered SLT practice models is a logical first step in supporting evidence-based decision-making using the strategies suggested by Archibald [32], as well as Cirrin and colleagues [33].

In previous work, we interviewed clinical managers and experienced S-LTs regarding their use of outcomes to guide practice during the transition to tiered services [38]. S-LTs identified outcomes as an important area for innovation and professional growth, and recognized that tiered services required new approaches [38]. However, in that study, we limited questions to current outcomes practices. We did not inquire about the outcomes that participants valued or wanted to achieve, although some clinicians did provide spontaneous responses in that regard. Subsequently, we conducted focus groups with caregivers, teachers, and clinicians to generate ideas about what outcomes were most valued by each participant group [40]. In that study, we explored valued and meaningful outcomes; however, we did not provide participants with the opportunity to react to ideas presented by other participants, nor did we attempt to fully synthesize the recommendations into a guiding framework. Therefore, in the present study, we attempt to explore and build consensus among these interested parties regarding the core outcomes for speech-language therapy service delivery models in schools.

The objective of this study was to develop a guiding framework for a Core Outcome Set for school-based speech-language delivery models by combining the perspectives of members of three key interested parties: family members of children with speech, language, or communication needs, teachers, and school-based S-LTs.

## Methods

We constructed a guiding framework for a COS using group concept mapping (GCM). In the present study, we focused on conceptual outcomes to inform a final COS, suggesting these outcomes as a guiding framework. In GCM, knowledge from groups with different perspectives on a topic is integrated using qualitative and quantitative methods, and then this combined knowledge is represented visually [41]. GCM is a participatory, mixed methods technique [42] that has been used successfully by other teams to construct frameworks for guiding measurement and evaluation with the input of multiple perspectives [41, 43], including in COS development [44, 45]. GCM is an elicitation approach that can identify important outcomes to guide research and evaluation [46, 47] by combining the stated perspectives of interested parties. This method can efficiently refine the data and clarify key points of agreement or disagreement [43, 44].

### Ethics

All methods and materials for this study were reviewed by the Hamilton Integrated Research Ethics Board (project number: 13906), which is affiliated with McMaster University, as well as all the research ethics committees of all partnering school boards. Methods and materials followed these reviewed ethical guidelines and regulations. All participants provided written informed consent prior to completing any research tasks.

### Sampling

We used purposeful sampling [48] followed by snowball sampling [48] to recruit representatives for each interested party. First, we purposefully recruited those who we considered to be likely to have relevant knowledge due to their extensive personal, clinical, or professional experience with tiered services. We assumed that families, educators, and S-LTs would require representation in the study as a starting point for our sampling strategy. Then, we asked newly enrolled participants who else they perceived as substantially affecting or being affected by school-based speech and language services. This snowball technique is recommended in participatory studies involving numerous groups to ensure that the participants best represent the social context of the problem under consideration [49, 50].

### Participants

We recruited 22 participants who completed at least one step of data collection, which is well within the suggested participant range for GCM studies [43]. Eleven were S-LTs affiliated with a school board; six were teachers; and five were family members of a child with experience receiving speech-language services in schools. All participants resided in the province of Ontario, Canada, in a locale where tiered services were being offered via school-based S-LTs. Tiered models are a common type of self-reported service delivery model used in the province [28]. Given our intended purpose of ultimately building a COS for tiered, school-based services in this context, generalizability beyond Ontario was not a specific goal of the study. Additionally, the reorganization of school-based services in the province is on-going [28] and may be considered a sensitive topic. Consequently, we followed best-practice recommendations [51] and did not collect additional demographic information on participants beyond their participant group. Not all participants completed all tasks, and so we specify the number of participants included in each of the analyses.

### Materials

In GCM studies, the materials consist of statements or ideas which are then analyzed and evaluated by participants. Participant responses are then aggregated to yield study results. In the present study, as we ultimately sought to construct a COS for speech-language service delivery models in schools, we used *indicators* as the statements. Indicators were generated from a summative content analysis [52] of 14 interviews from a previous study [40], where participants were asked about what outcomes of speech and language services were most important or meaningful. In this content analysis, interviews were open coded with the data analyst staying close to the data [53] and using the terminology of participants [52] to tag potential indicators. Several rounds of peer debriefing [53] were conducted to review indicators and collapse categories down to a manageable level. An original list of 146 potential indicators was ultimately condensed to 103. See Online Supplementary Material for a list of the indicators that served as the materials for this study.

### Procedures

Participants were sent an invitation with an anonymized link to access *groupwisdom* [54], a software platform supporting digital group concept mapping. Participants first indicated whether they identified primarily as a family member, S-LT, or teacher. Next, participants sorted indicators into virtual piles and provided suggested names for each grouping of statements. The instructions were to sort the statements into piles that made sense to each participant, putting ideas that were more similar together. The order of statements was randomized for each participant to protect against the ordering contaminating the similarity sorting. Participants then rated each indicator for its meaningfulness or importance and then its feasibility on five-point scales, with five indicating the most importance or feasibility rating and one the lowest rating. The specific questions were as follows:

1. *How important or meaningful do you think this idea is?*
2. *How feasible or doable would this idea be in day-to-day work?*

Although each task began automatically upon completion of the previous activity, participants were not required to complete all tasks, and so participant numbers were not fully consistent across each step and are reported separately below. These three tasks (demographic question, sorting and naming, and rating) represented all participant data collection procedures within this study.

### Data analysis and modelling

#### Creating the cluster map

Sorting data was converted into a similarity matrix, identifying the number of participants who placed two statements into the same pile [43]. Then, multidimensional scaling was applied to the similarity matrix to yield a point map of all indicators, with the distance between each pair of indicators representing the probability of being sorted together [43]. This assigned each statement coordinates on a two-dimensional plane. We used the observed stress value for our multidimensional scaling solution to assess the goodness-of-fit of the solution to the underlying similarity matrix, targeting a value of ≥ 0.365 following methodological guidelines [43, 55]. Data from all participants were carried forward into the cluster analysis. This included participants who appeared to sort the data differently from the majority (specifically by grouping statements by current implementation in practice rather than the intended conceptual grouping around similarity in ideas). With this approach, we maximised the data that were included without unduly impacting the stress value.

**Fig. 1:**
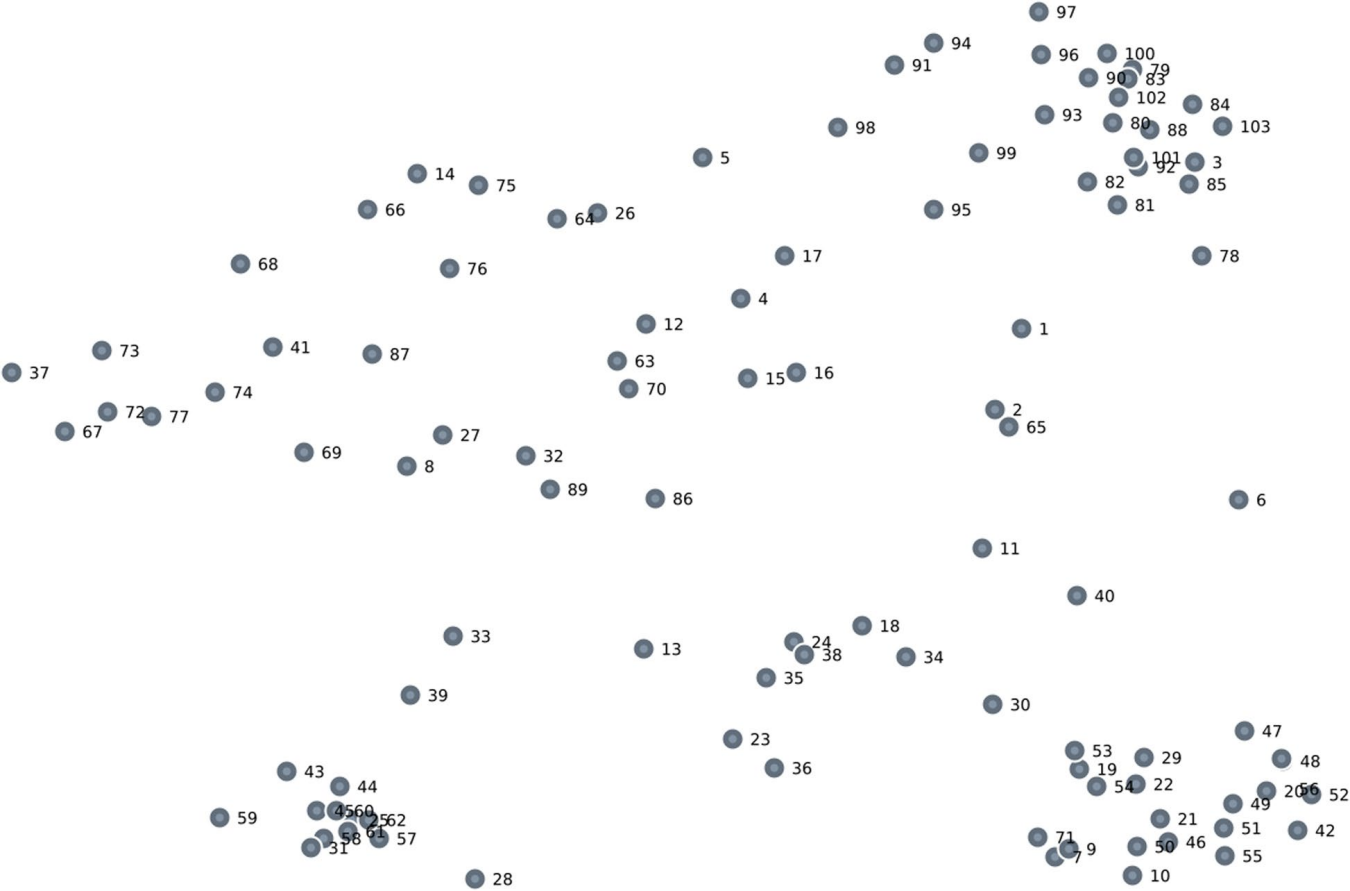
Point map showing accepted multidimensional scaling solution

Subsequently, hierarchical cluster analysis was performed using Ward’s algorithm [56, 57] on these coordinates, yielding a *cluster map* with similar outcome ideas amalgamated into two-dimensional polygons. The first author considered a range of cluster solutions, starting at 18 (the largest number of piles used by any participant in the sorting task) and removed one cluster at a time iteratively until we reached three clusters (identified as smallest possible number based on visual inspection of the results, see Fig. 1 in results). Each cluster solution was evaluated for interpretability and meaningfulness by the first author until a smaller set of potential cluster solutions was selected. This list of finalists was discussed within the research team (PC and WC) in peer debriefing, and the final cluster solution was selected through team consensus. The first author then calculated the mean ratings for all participants for each cluster.

#### Comparing group perspectives

The first author calculated separate estimates for mean importance and feasibility for each participant group, using visualizations of these estimates to identify potential areas of disagreement or differential prioritization among the interested parties. We looked at the overall cluster ratings, as we were interested in how participants evaluated each outcome for importance and feasibility, rather than each indicator. Indicators were assessed separately (see next section).

#### Evaluating indicators

To determine which indicators best represented their respective outcome and to identify the most promising indicators for immediate implementation, we inspected the mean ratings of each indicator within its assigned cluster. Indicators that were rated above average in importance and feasibility were considered to be recommended for implementation in practice. Indicators that were above average in importance but below average in feasibility were considered promising, needing future research to investigate how these indicators could be targeted. Indicators with below average importance ratings for their cluster were discarded as not being of sufficient relevance to be included in the COS guiding framework.

#### Constructing the guiding framework

Upon completing all the above analyses, we constructed the final COS guiding framework by including all the clusters from the final cluster solution representing each outcome. We then appended both recommended and promising indicators to provide indications of how these outcomes could be assessed or measured within research and practice. Finally, a tabular visualization of the guiding framework was created to assist in interpretation and knowledge mobilization.

## Results

In this study, we aimed to construct a guiding framework for a COS for school-based speech-language services using GCM to combine the perspectives of interested parties.

### Sorting

#### Multidimensional scaling of sorting data

Twenty participants completed the sorting step. Then, the research team transformed the sorting data into a two-dimensional point map using multidimensional scaling. The solution is represented in Fig. 1, where the distances between points indicate the probability of two indicators being sorted together. The closer two points are on the map, the more likely the two indicators were to be sorted together by participants. A visual inspection of the point map strongly suggests a minimum of three clusters (one in the upper right corner as well as in both lower corners).

To evaluate the validity of the scaling solution, we compared the observed stress value to values obtained in previous GCM studies and to stress value cut-offs suggested in the methodological literature [55, 58]. A lower stress value indicates that the multidimensional scaling solution better reflects the original data. Our observed value (0.24) was below the mean of 0.28 (95% confidence interval: 0.27-0.29) for GCM studies [55], and well below the cut-off value of 0.39 indicating a one percent chance that the solution reflects only random information with no underlying structure [58]. Consequently, we accepted this solution and continued to subsequent steps in the analysis.

#### Cluster analysis of sorting data

**Fig. 2:**
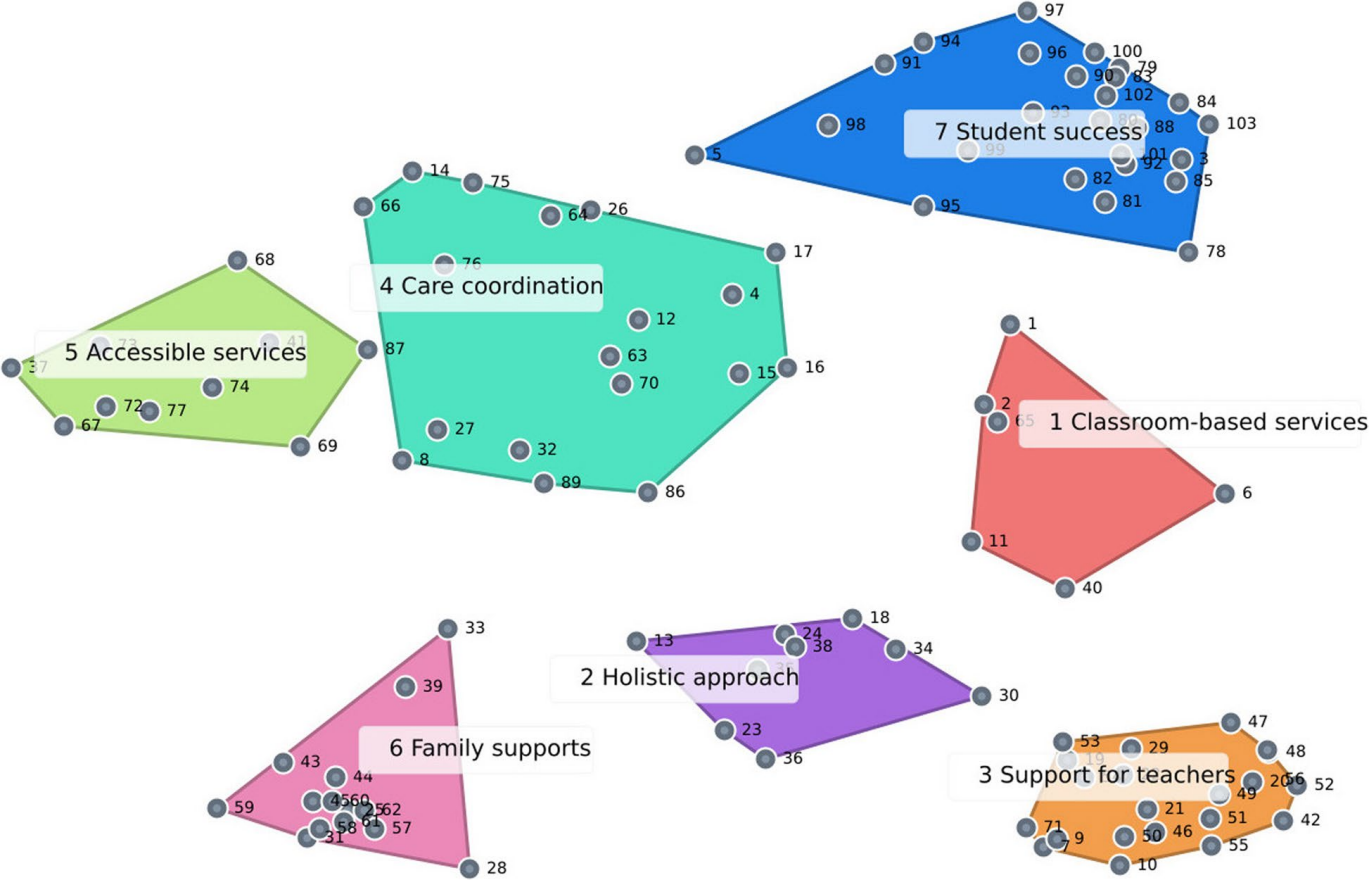
Cluster map for seven-cluster solution

We then analysed the scaled data using hierarchical cluster analysis [41, 43], which is an appropriate clustering algorithm for such data [57]. We estimated 16 cluster solutions (18 to 3 clusters) and reviewed all cluster solutions beginning with the 18-cluster solution and removing one cluster at a time and inspecting quantitative and qualitative aspects of each solution. We narrowed the cluster solutions to 6-8 based primarily on qualitative judgements about the conceptual clarity and consistency. Particularly, the first author focused on whether a clear conceptual separation could be found between all clusters. If no clear definition of all clusters could be found, the first author continued combining clusters and re-evaluating the conceptual clarity of the smaller number of groupings. These solutions were discussed in greater detail in multiple peer debriefing meetings (PC and WC). Ultimately, we selected a seven-cluster solution. We used names generated by participants to name each cluster, with minor editing sometimes required to expand abbreviations and rephrase into parallel structures. These names were also discussed and minorly revised in peer debriefing meetings. The revised names were then applied to the cluster map (Fig. 2).

Cluster one was *classroom-based services*. The indicators (*n* = 6) in this cluster were associated with personalization of recommendations and goals to match the curriculum and be feasible in and suitable to busy classrooms. Cluster two was a *holistic approach* (*n* = 9). This cluster focused on the overarching orientation and philosophy of speech-language services in schools, including having open, trusting, and collaborative relationships and fostering an inclusive school culture. Cluster three was *support for teachers* (*n* = 21), which was focused on the development of teacher capacities to support children with communication needs. This cluster also included the ability of teachers to freely access and consistently communicate with the S-LT. Cluster four was *care coordination* (*n* = 18). This cluster focused on how S-LTs could direct and facilitate access to appropriate services, and to match recommendations and services to students’ needs. Cluster five was *accessible services*, which included indicators (*n* = 10) of service timeliness and the adequacy of resources to provide responsive services, as well as streamlining the steps and procedures required before supports can be accessed. Cluster six was *family supports* (*n* = 14), which focused on family experiences and satisfaction, as well as their engagement with the decision making and implementation of supports. Cluster seven was *student success* (*n* = 25), where children are included in the school, are engaged in learning, are happy and thriving, and developing their functional communication skills.

#### Rating

Eighteen participants provided rating data. These included: eight S-LTs, six teachers, and four family members. Pooled mean ratings for all indicators within a cluster are reported in the following sections. Individual indicator ratings can be found in the Online Supplementary Material.

#### Overall ratings

**Fig. 3:**
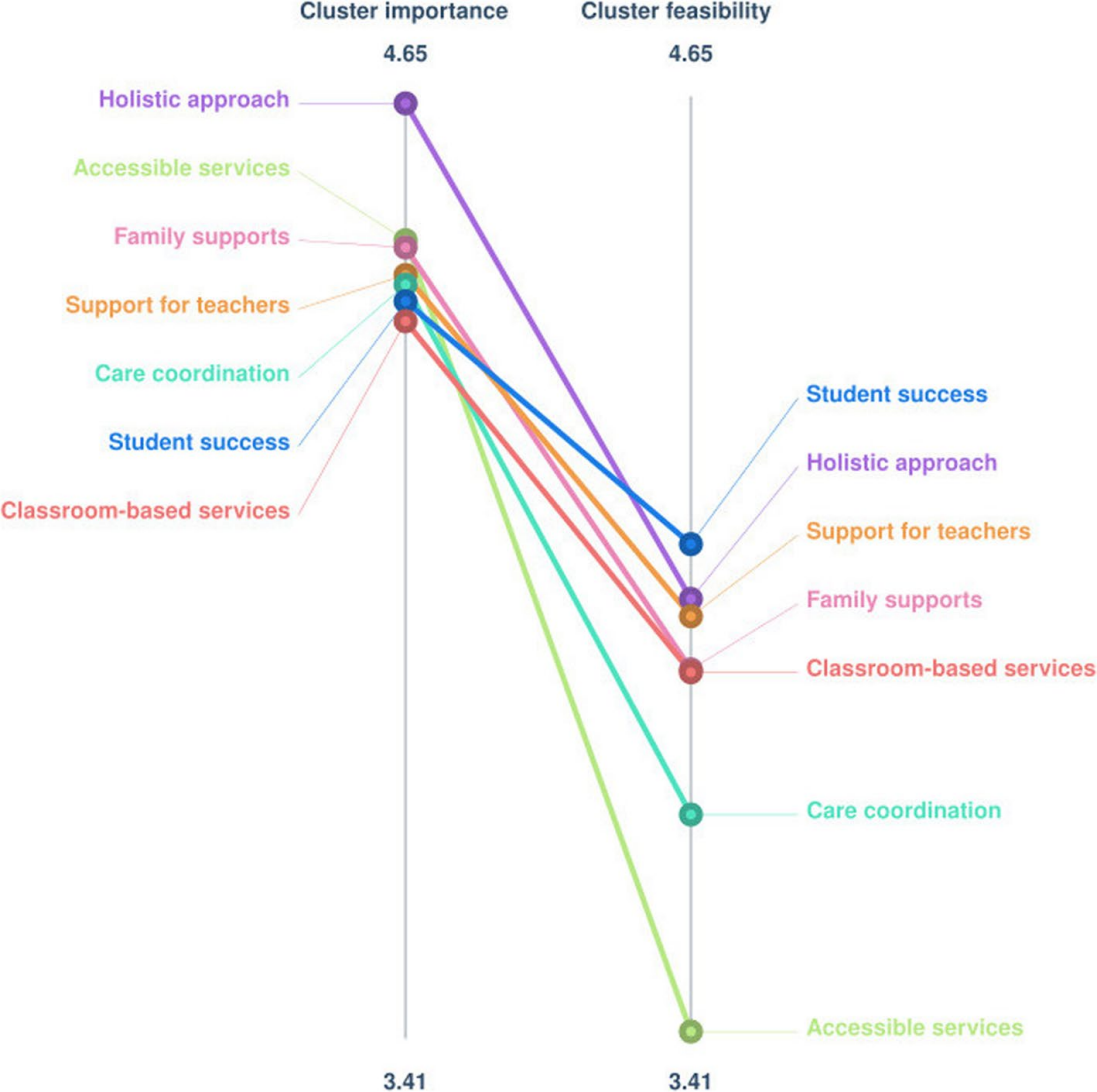
Pattern match graph of overall importance and feasibility ratings for each outcome on absolute scale

Combining ratings from all participant groups, all outcomes were rated highly for importance (above four on a five-point scale). Participants rated the feasibility of assessing these outcomes somewhat lower (from 4.06 to 3.41 on a five-point scale). This is illustrated in Fig. 3.

#### Comparison of group ratings

**Fig. 4:**
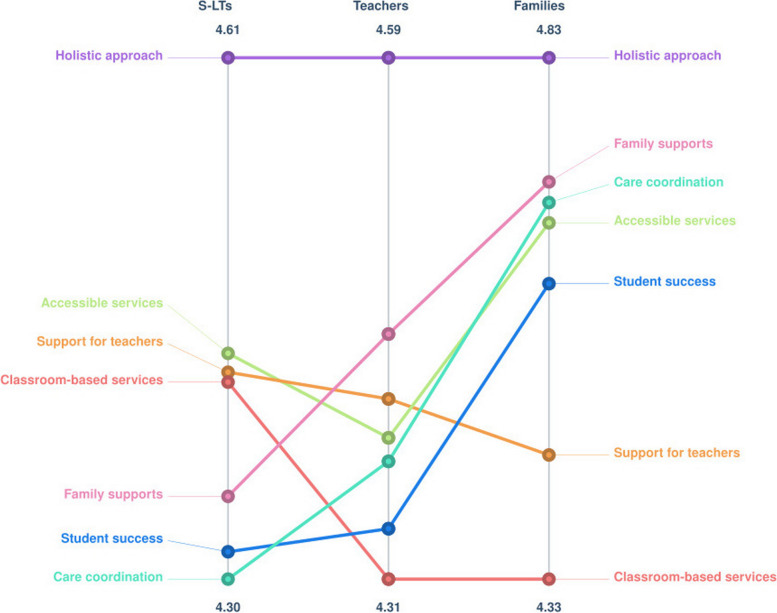
Pattern match graph for outcome importance by participant group on a relative scale

Participant groups rated most outcomes differently for importance as can be seen in Fig. 4. It is important to note that these ratings are considered relative rather than absolute [41], and so it is the ordering rather than the magnitude that is of note. One notable finding was the consensus that a *holistic approach* was the most important outcome across all participant groups.

**Fig. 5:**
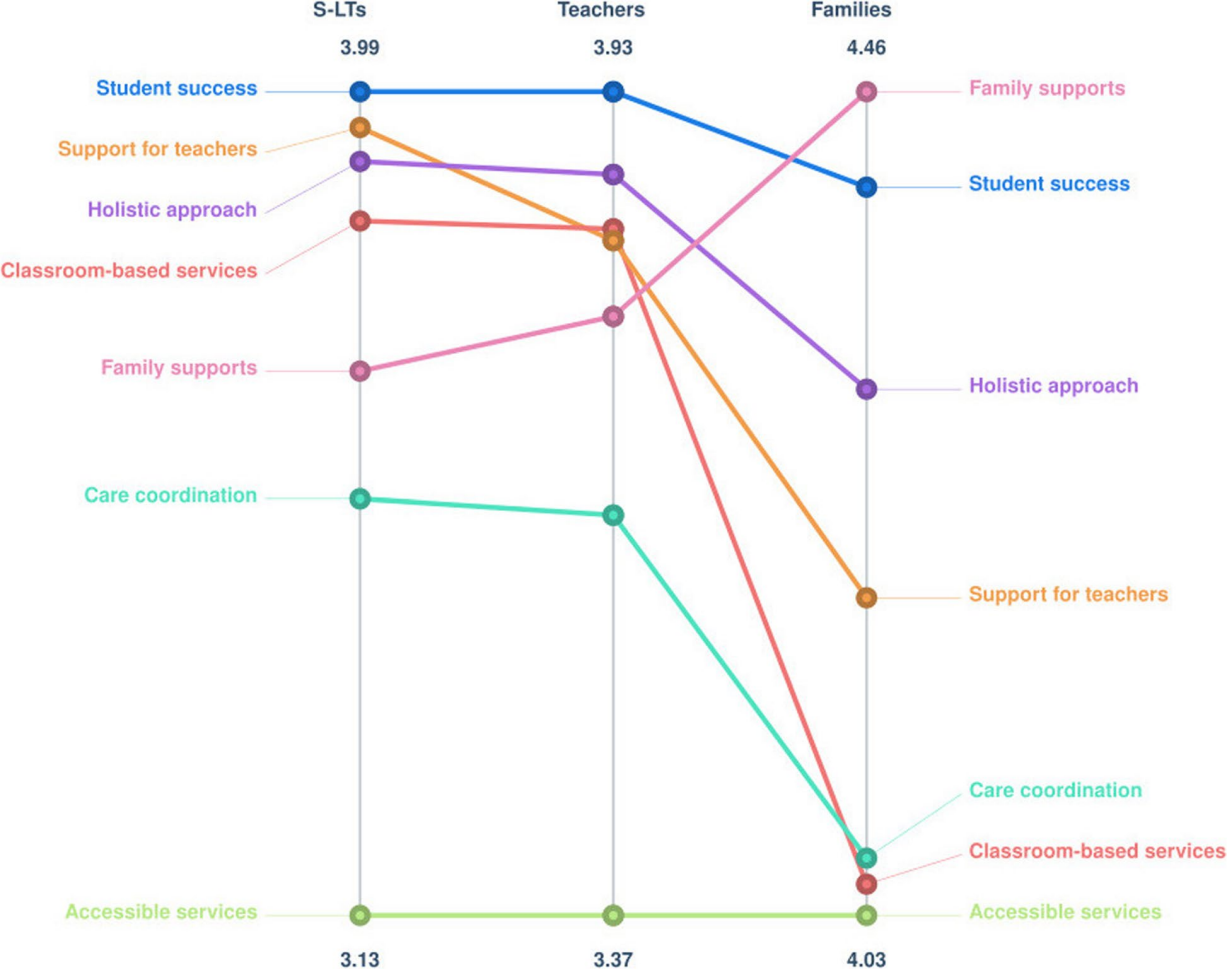
Pattern match graph for outcome feasibility by participant group on a relative scale

A similar pattern was observed for feasibility ratings, with different average ratings across most outcomes. However, there was universal agreement that *accessible services* was the least feasible outcome to achieve. See Fig. 5 for a visual representation.

Table 1 summarizes our findings, as a guide to future research and evaluation work. We included each core outcome, accompanied by the indicators that participants rated as having above-average importance. Those on the left and in bold are those that were also rated above the mean on feasibility. We called these recommended indicators. In contrast, indicators on the right and in italics were rated below average on feasibility, which we called promising indicators. Promising indicators may require additional consideration or exploration before they can be considered as outcomes for research or clinical evaluation activities.

**Table 1:** Guiding framework for school-based speech-language services with recommended and promising indicators

***Outcome***	***Recommended indicators*** ***(rated important and feasible)***	***Promising indicators*** ***(rated important but less feasible)***
***Classroom-based services***	**children’s goals are personalized** **children’s goals are constantly updated to reflect progress** **SLP supports complement coursework and classroom learning**	*SLP supports and techniques work in a busy classroom environment* *each school has a consistent, assigned SLP*
***Holistic approach***	**the school works as a team to support each child’s communication development** **the school is an inclusive place that supports students with all needs**	*each school receives SLP services tailored to the school’s needs* *the school has the staff needed to support the needs of all children*
***Support for teachers***	**teachers pinpoint specific student needs in collaboration with SLP** **teachers can access SLPs directly with questions or concerns** **teachers use SLP strategies and recommendations in the classroom** **teachers develop strategies to support communication development in the classroom** **teachers develop skills and techniques to support specific students** **teachers can use techniques independently after concrete demonstration, modelling, or training** **teachers feel confident in their abilities to support students** **teachers learn about their students’ specific communication needs**	*teachers are provided with the right equipment to support their students* *teachers can access professional development opportunities via the SLPs*
***Care coordination***	**children with greater needs spend more time with SLPs and in SLP programming** **SLP programming for each student is tailored and individualized** **SLP recommendations and suggestions are not overly complicated** **SLPs advocate to meet children’s needs** **children who need SLP supports are identified very early** **resources are carefully matched to children’s needs and skills** **communication challenges are identified and not confused with behavioural concerns**	*children’s supports are carefully matched with their needs* *children receive consistent, frequent, individualized classroom-based supports* *all children with needs receive services, and not just a subset* *children do not need a formal diagnosis to access supports* *supports are implemented very early, near when children enter school*
***Accessible services***	**SLPs can access specialty training to support children with unique needs** **resources are allocated to provide maximum impact**	*SLP supports and services are appropriately funded* *waitlists are minimized* *gatekeeping and obstacles to supports are removed or reduced*
***Family supports***	**parents hear a consistent and unified message from teachers and SLPs** **families know about all SLP recommendations** **families feel included in decision-making** **families feel supported by the school professionals**	*appropriate services are fully supported by administration and policy* *families learn SLP strategies to use at home*
***Student success***	**children are more confident and independent** **children enjoy the supports they receive from SLPs** **children who receive supports do not feel different or singled out** **children communicate more easily and willingly in class** **children are able to bring together multiple skills to communicate, read, and write** **children engage socially with their classmates** **children find SLP supports helpful** **children settle in and become more comfortable in the classroom** **children use strategies and techniques taught by SLPs** **children have better self-esteem** **children can eventually participate in society and gain employment** **children do not feel pressured or intimidated by SLP activities** **children have greater quality of life** **children understand others’ communication** **others understand the child’s communication**	*children demonstrate improvement on assessments of specific communication skills* *children learn how to include their peers with communication difficulties*

The local term *SLP* (speech-language pathologist or speech-language pathology) was used in the statements

## Discussion

In this study, we constructed a guiding framework for COS for school-based SLT service delivery models through GCM, using clusters to represent core outcomes. We identified seven core outcomes: a holistic approach, accessible services, family supports, support for teachers, care coordination, student success, and classroom-based services. All outcomes were rated as highly important, and no outcome was consistently rated as least important by all three participant groups. Consequently, we retained all seven outcomes in the guiding framework. There were diverse opinions among participant groups regarding the importance and feasibility of each outcome, suggesting different groups had different priorities. However, all participant groups rated achieving a holistic approach to services as the most important outcome, indicating a striking consensus regarding the importance of centring collaboration and inclusive culture and practice within school-based speech and language services. The outcome of accessible services was rated as the least feasible to achieve by all participant groups, again reflecting an area of substantial agreement. Previous research has identified waitlists and other service barriers as a major problem to address [59–61]. Our results support this finding and highlight the need for creative solutions to make services accessible to all children. Interestingly, the participants rated speciality training for S-LTs and the ability to distribute SLT resources according to impact as highly feasible. These suggestions provide guidance for future work.

In previous, related work [38, 40], we have noted a focus, particularly on the part of professionals, on *processes* when asked about *outcomes*. In other words, they discussed the ways in which they aimed to achieve their targeted outcomes. This was unsurprising, as separating the structures, processes, and outcomes of care can be conceptually difficult [2]. Similarly, in this study, we noted that the core outcomes included essential processes to achieve distal outcomes. Particularly, we interpreted the three centrally located core outcomes on the cluster map (i.e., holistic approach, classroom-based services, and care coordination) as representing essential processes. In contrast, the four corner clusters (accessible services, family supports, supports for teachers, and student success) represented the ultimate outcomes of services. Tracking essential practices to achieving desired outcomes is justified within quality appraisal [1, 2] and evaluation [62, 63] for health services. Consequently, we included these process-focused outcomes, as our participants’ perspectives clearly supported these processes as essential aspects of high-quality speech-language services in schools.

The absence of the core outcomes identified in the present study in the literature to date is stark, empirically confirming previous observations in the conceptual literature [34], as well findings from a comparison of SLT and educational research outcomes [35]. Although the reviews by Cirrin et al. [33] and Archibald [32] targeted broader outcomes such as impacts on referral rates and teacher and parent use of language facilitation techniques, the literature located by those reviews report only student-level clinical outcomes such as standardized assessment battery scores, language sample analysis measures, and bespoke skill assessments. A similar focus on student-level clinical outcomes is evidenced in the review by Ebbels and colleagues [30], where SLT inventions such as professional development for teachers are only considered in so far as they produced measurable changes in children’s clinical assessments, such as vocabulary or literacy skills. Not only does this focus on clinical outcomes in the primary literature exclude core outcomes identified in this review, such as supports for families or timely and easy-to-access referral systems, but it also excludes broader conceptualizations of student success, such as quality of life, student perceptions of inclusion within the school community, or inclusion in social and academic life. These broader conceptualizations were exclusively identified as important student-level outcomes in a previous study [40], as well as with a similar study investigating outcomes valued by families, teachers, and children with communication disorders [64]. They are also more similar to the COS developed by Morris and colleagues [18] for children with a neurodisability and to the outcomes present in educational research [35] than they are with the outcomes typically found in research studies within speech-language therapy. Of particular note, the school-based S-LTs who participated in this study seemed to value the types of outcomes that predominant in the educational literature [see 35], rather than those common in the SLT literature. This highlights the need for such a guiding framework, and an expansion by SLT research into broader service outcomes.

Our guiding framework does not exclude clinical outcome measures. The literature on COS has consistently supported the inclusion of intervention-specific outcomes in addition to the appropriate COS [14, 16]. However, research into school-based service delivery models must expand to collect information about how the service affects the school community and its functioning. Additionally, student-level outcomes should be expanded to include assessments of participation and well-being. These recommendations echo those from reviews of other areas of paediatric speech-language therapy [65, 66], which have called for inclusion of well-being, participation, and experience measures in SLT research. These types of student-level outcomes do not appear to have been collected in previous school-based SLT research according to reviews [30, 32, 33]. They should be included in future work. It is also essential to include the child’s individual voice in determining what these student-level outcomes look like, as *success* may look different to different children. Imposing a standardized conceptualization of success would undermine the goal of responsive and individualized services.

To use this guiding framework in practice to evaluate or improve school-based services, there may be additional challenges and opportunities. First, many of the indicators listed in the present results require data beyond what might be collected by the S-LT. Rather, these data may exist in administrative databases and may be best collected or accessed by other professionals, such as school leadership. This echoes the words of a participant in a previous study [38] that outcomes data in schools are available, but not in the form that is traditionally considered clinical outcomes data. Additionally, because system-level data are necessary for this guiding framework, collaboration within the SLT team as well as support and active involvement of school administration will be required. This is consistent with previous research indicating that SLT collaboration is an important support for the meaningful use of outcomes [38] and that administration and leadership will need to value outcomes and successful SLT practice if meaningful changes are to be made [28, 38]. With the complexity and diversity of the data required to address the seven core outcomes in this set, it would likely be necessary to mobilize substantial resources and implement an outcomes data management strategic plan, with guidance from important school community members such as S-LTs and family members. This institutional effort should be further supported by systems-informed thinking [4–6] to ensure that outcome indicators are used to meaningfully inform care.

### Limitations

This study has four major limitations. First, we limited the scope of this study to a narrow geographic area. Consequently, we cannot be sure that a guiding framework for a COS developed elsewhere would not include different outcomes. However, this limitation is shared with most COS development projects [19]; further, simultaneously developed COS in Sri Lanka and the United Kingdom have produced similar results [19]. Consequently, we recommend that COS development for other locales use the results of the present study as a starting point to expedite and accelerate their own work. Beyond this, we did not prioritize an equity lens to approach recruitment or data interpretation. Consequently, we do not know what additional richness and nuance diverse perspectives would have brought to the results.

Second, rating data are based on a restricted scale collected from a small sample. Consequently, the mean estimates are statistically noisy and only broad patterns should be interpreted from this data. It is possible that with a larger number of respondents or a more sensitive measurement approach that the ordering and the magnitude of rating differences would be more stable. For these reasons, we strongly urge against overinterpretation of these estimates.

Third, although we used an appropriate multidimensional scaling solution [55, 58] and cluster analysis algorithm [57], it is possible that indicators could be interpreted conceptually as belonging to more than one cluster. In other words, the algorithm we used imposes a single membership model, eliminating the possibility for overlap and multi-class membership. Therefore, it is important to focus on the overall conceptual focus of each outcome, rather than on the classification of any particular indicator. While it is possible to manually recategorize indicators into other clusters [43], we chose to not intervene in the results of the multidimensional scaling or cluster analysis. Instead, we have chosen to reserve robust conceptual clarification for future work, where we hope to explore the most appropriate assessment methods for each outcome. This is particularly important due to the final major limitation of this study: the absence of students’ voices.

Last, we did not include students with speech, language or communication needs in the present study due to our desire to use a highly structured elicitation technique, which young people would likely find challenging [67]. Although this technique has been used with youth and adolescents [67], it required substantial modifications for this purpose, and has not been evaluated for suitability for younger school students with various accessibility needs. We suggest that these findings be further explored conceptually with methods more friendly to diverse participants, much like those used by Gallagher and colleagues [64]. In that study [64], the researchers were able to explore the outcomes that young students valued and how these were consistent with the outcomes identified by adults, while offering a nuanced interpretation of each outcome from the student’s perspective. We suggest that such an exploration of students’ interpretations should be considered for future research as an essential part of robustly conceptualizing each of these outcomes.

## Conclusions

In summary, we developed a guiding framework for a COS for school-based speech-language service delivery in Ontario schools. Having identified the key outcomes of these services, this work enables future evaluation and improvement work [39] when combined with an appropriate theory [63, 68]. Specific to those working in schools within a tiered model of service delivery, the initial programme theory developed by VanderKaay and colleagues [36] may be of particular interest. Tiered models are widely used internationally [23, 24, 27–29, 31], and this programme theory has been developed specifically for these practice contexts and models. Consistent with COS recommendations [14–16], we endorse incorporation of these core outcomes into university and school-based research activities where possible, complementing other outcomes specific to the intervention or service changes under investigation. Further work is required, however, to identify the most appropriate ways to assess or measure these outcomes, as the forms of some indicators provided in the present framework are not immediately measurable. Judicious selection from this guiding framework may therefore be necessary in the interim. We recommend exploring aspects of these core outcomes qualitatively, making use of methodological advancement into mixed-methods clinical trials [69, 70] or program evaluations [39, 62, 63] to determine the impacts of service design and delivery within schools.

### Supplementary Information

**Supplementary Material 1.** 

## Data Availability

The data generated and analysed in the present study are available, with exceptions, through the following link: https://doi.org/10.17605/OSF.IO/7YA62. Participant demographic information has not been shared due to the potentially sensitive nature of the issue under consideration within the participants’ province of residence.

## Abbreviations

COS: Core outcome set
S-LT: Speech-language therapist
S-LTs: Speech-language therapists
SLT: Speech language therapy
