# Supplementary material for: Core outcomes for speech-language services in Ontario schools: a group concept mapping study and guiding framework
Source: BMC Health Serv Res. 2024 Mar 16;24:347. doi: 10.1186/s12913-024-10821-7 (PMC10943816; doi:10.1186/s12913-024-10821-7)
Supplement: Supplementary file 1 — Supplementary Material 1. [file 12913_2024_10821_MOESM1_ESM.docx]

Online Supplementary Material

Complete list of outcomes identified in open coding with mean ratings across participant groups

Note: SLP here can refer to either speech-language pathologist or speech-language pathology depending on syntactic context.

| **ID** | **Indicator** | **Mean importance rating** | **Mean feasibility rating** |
| --- | --- | --- | --- |
| 1 | children's goals are personalized | 4.56 | 4.22 |
| 2 | children's goals are constantly updated to reflect progress | 4.50 | 4.00 |
| 3 | children are in the classroom and learning with their peers | 4.22 | 3.94 |
| 4 | children's supports are carefully matched with their needs | 4.72 | 3.61 |
| 5 | children are not pulled out of class to receive SLP services | 3.00 | 2.72 |
| 6 | SLP supports complement coursework and classroom learning | 4.39 | 3.89 |
| 7 | teachers can access SLP advice even before a formal referral is made | 4.33 | 3.78 |
| 8 | SLP suggestions and advice are chunked into manageable bits | 4.39 | 4.50 |
| 9 | teachers are provided with the right equipment to support their students | 4.56 | 3.83 |
| 10 | teachers can access different types of SLP supports as needed | 4.39 | 3.83 |
| 11 | SLP supports and techniques work in a busy classroom environment | 4.56 | 3.72 |
| 12 | children with greater needs spend more time with SLPs and in SLP programming | 4.56 | 3.83 |
| 13 | each school receives SLP services tailored to the school's needs | 4.72 | 3.61 |
| 14 | all children can access evidence based SLP supports and programming | 4.22 | 3.33 |
| 15 | children received tailored supports very soon after entering school | 4.28 | 3.33 |
| 16 | SLP programming for each student is tailored and individualized | 4.50 | 4.00 |
| 17 | children receive consistent, frequent, individualized classroom-based supports | 4.44 | 3.33 |
| 18 | families and teachers provide open and honest feedback to SLPs | 4.56 | 4.22 |
| 19 | teachers pinpoint specific student needs in collaboration with SLP | 4.71 | 4.11 |
| 20 | teachers can access professional development opportunities via the SLPs | 4.44 | 3.67 |
| 21 | teachers can ask SLPs questions directly and consistently | 4.33 | 3.89 |
| 22 | teachers provide feedback on what is working and what is not | 4.39 | 4.17 |
| 23 | SLPs know and regularly communicate with other team members, such as teachers | 4.56 | 4.39 |
| 24 | SLPs collaborate and problem solve directly with other staff (teachers, other health professionals, etc...) | 4.61 | 4.17 |
| 25 | families are clearly, often, and proactively informed regarding child progress | 4.44 | 3.67 |
| 26 | children are followed consistently by the same professionals | 4.00 | 2.94 |
| 27 | SLP recommendations and suggestions are not overly complicated | 4.44 | 4.33 |
| 28 | parents hear a consistent and unified message from teachers and SLPs | 4.50 | 3.94 |
| 29 | teachers can access SLPs directly with questions or concerns | 4.56 | 4.06 |
| 30 | the school works as a team to support each child's communication development | 4.72 | 4.22 |
| 31 | parents communicate with the school as a whole team, instead of needing to speak with each professional separately | 4.00 | 3.44 |
| 32 | SLPs advocate to meet children's needs | 4.72 | 4.44 |
| 33 | appropriate services are fully supported by administration and policy | 4.72 | 3.78 |
| 34 | the school is an inclusive place that supports students with all needs | 4.72 | 4.11 |
| 35 | the school provides supports and accommodations to children proactively and inclusively | 4.61 | 3.94 |
| 36 | the school has the staff needed to support the needs of all children | 4.89 | 2.89 |
| 37 | SLP supports and services are appropriately funded | 4.83 | 2.83 |
| 38 | SLPs work as equal team members with other professionals | 4.50 | 4.33 |
| 39 | SLPs work as equal team members with caregivers and parents | 4.39 | 3.89 |
| 40 | each school has a consistent, assigned SLP | 4.50 | 3.61 |
| 41 | SLPs can access specialty training to support children with unique needs | 4.61 | 3.72 |
| 42 | teachers use SLP strategies and recommendations in the classroom | 4.67 | 4.11 |
| 43 | families can easily access information and assistance with navigating services in schools | 4.44 | 3.78 |
| 44 | families know about all SLP recommendations | 4.61 | 4.06 |
| 45 | families know about all options to support their child | 4.44 | 4.00 |
| 46 | teacher's needs and questions are met quickly and consistently | 4.35 | 3.56 |
| 47 | teachers develop strategies to support communication development in the classroom | 4.50 | 4.00 |
| 48 | teachers develop skills and techniques to support specific students | 4.50 | 4.28 |
| 49 | teachers can distinguish between developmental concerns and normal communication development | 4.33 | 3.61 |
| 50 | teachers know where and how to access various supports for their students | 4.39 | 4.06 |
| 51 | teachers can use techniques independently after concrete demonstration, modelling, or training | 4.56 | 4.00 |
| 52 | teachers feel confident in their abilities to support students | 4.44 | 4.11 |
| 53 | teachers learn about their students' specific communication needs | 4.67 | 4.17 |
| 54 | teachers enjoy using the techniques and supports recommended by SLPs | 4.00 | 3.72 |
| 55 | teachers enjoy collaborating with SLPs | 4.11 | 4.22 |
| 56 | teachers can use previously learned strategies with other children | 4.39 | 4.47 |
| 57 | families feel included in decision-making | 4.56 | 4.06 |
| 58 | families are oriented to resources and opportunities in the community to support their child | 4.33 | 3.89 |
| 59 | families know what to expect from SLP services | 4.39 | 4.11 |
| 60 | families learn SLP strategies to use at home | 4.72 | 3.78 |
| 61 | families receive consistent, regular updates on children's progress | 4.33 | 3.89 |
| 62 | families feel supported by the school professionals | 4.56 | 4.22 |
| 63 | children who need SLP supports are identified very early | 4.72 | 3.83 |
| 64 | fewer children need intensive SLP services in the long term | 4.06 | 3.72 |
| 65 | fewer children with normal communication abilities are referred to SLPs | 3.67 | 3.89 |
| 66 | all children are screened for communication difficulties early on | 3.83 | 3.11 |
| 67 | referrals for SLP services are highly accurate, with few false negatives or positives | 4.00 | 3.22 |
| 68 | SLPs dedicate more time to children with the greatest needs | 4.22 | 3.94 |
| 69 | referrals are quickly addressed and recommendations rapidly made | 4.44 | 3.50 |
| 70 | resources are carefully matched to children's needs and skills | 4.50 | 3.89 |
| 71 | teachers know how to make accurate referrals, including appropriate procedures | 4.28 | 3.61 |
| 72 | resources are allocated to provide maximum impact | 4.65 | 3.72 |
| 73 | waitlists are minimized | 4.56 | 3.00 |
| 74 | wait times are reduced | 4.44 | 3.33 |
| 75 | all children with needs receive services, and not just a subset | 4.44 | 3.28 |
| 76 | children do not need a formal diagnosis to access supports | 4.50 | 3.56 |
| 77 | gatekeeping and obstacles to supports are removed or reduced | 4.50 | 3.22 |
| 78 | children are meeting goals or expectations | 4.28 | 4.11 |
| 79 | children are more confident and independent | 4.78 | 4.28 |
| 80 | children participate in all aspects of their school day | 4.22 | 3.59 |
| 81 | children enjoy the supports they receive from SLPs | 4.39 | 4.28 |
| 82 | children who receive supports do not feel different or singled out | 4.44 | 4.17 |
| 83 | children communicate more easily and willingly in class | 4.72 | 4.22 |
| 84 | children show progress on their report cards | 3.72 | 3.67 |
| 85 | children demonstrate improvement on assessments of specific communication skills | 4.39 | 3.83 |
| 86 | communication challenges are identified and not confused with behavioural concerns | 4.50 | 3.94 |
| 87 | unusual or uncommonly seen conditions are identified for further investigation | 4.44 | 3.56 |
| 88 | children are able to bring together multiple skills to communicate, read, and write | 4.61 | 4.22 |
| 89 | supports are implemented very early, near when children enter school | 4.56 | 3.56 |
| 90 | children engage socially with their classmates | 4.67 | 4.22 |
| 91 | children find SLP supports enjoyable | 4.33 | 4.39 |
| 92 | children find SLP supports helpful | 4.39 | 4.33 |
| 93 | children settle in and become more comfortable in the classroom | 4.56 | 4.11 |
| 94 | children use strategies and techniques taught by SLPs | 4.44 | 4.22 |
| 95 | all children show progress and gains from whole class SLP supports | 3.94 | 3.83 |
| 96 | children have better self-esteem | 4.78 | 4.28 |
| 97 | children can eventually participate in society and gain employment | 4.67 | 4.11 |
| 98 | children do not feel pressured or intimidated by SLP activities | 4.50 | 4.39 |
| 99 | children find SLP supports consistent and know what to expect | 4.22 | 4.00 |
| 100 | children have greater quality of life | 4.72 | 4.39 |
| 101 | children learn how to include their peers with communication difficulties | 4.67 | 4.00 |
| 102 | children understand others' communication | 4.44 | 4.11 |
| 103 | others understand the child's communication | 4.59 | 4.11 |
